# Mother’s Infant and Young Child Feeding (IYCF) knowledge improved timely initiation of complementary feeding of children aged 6–24 months in the rural population of northwest Ethiopia

**DOI:** 10.1186/s13104-018-3703-0

**Published:** 2018-08-16

**Authors:** Gashaw Andargie Biks, Amare Tariku, Molla Mesele Wassie, Terefe Derso

**Affiliations:** 1 Department of Health Service Management and Health Economics, Institute of Public Health, College of Medicine and Health Sciences, University of Gondar, Gondar, Ethiopia; 2 Department of Human Nutrition, Institute of Public Health, College of Medicine and Health Sciences, University of Gondar, Po.box: 196, Gondar, Ethiopia

**Keywords:** IYCF knowledge, Complementary feeding, Dietary diversity

## Abstract

**Objectives:**

Appropriate complementary feeding is vital to reduce young child morbidity and mortality. However, it continues as sub-optimal in Ethiopia, and literatures are also scarce. Therefore, this study aimed to determine timely initiation of complementary feeding and associated factors among mothers with children aged 6–24 months in the rural population of northwest Ethiopia. In the community based cross-sectional study, data on child feeding practices, individual and household characteristics were collected in Dabat Demographic Surveillance System site, Dabat District, northwest Ethiopia from 01 May to 29 June 2015. The bivariate and backward stepwise multivariable statistical methods were carried out to identify factors associated with timely initiation of complementary feeding.

**Results:**

About 53.8% [95% CI 45.9, 61.7] and 4.6% [95% CI 1.3, 7.9] of children were found with timely initiation of complementary feeding and had minimum dietary diversity, respectively. The odds of timely initiation of complementary feeding was higher among mothers with medium [AOR = 2.34, 95% CI 1.54, 3.81] and high [AOR = 2.10, 95% CI 1.41, 3.87] mother’s IYCF knowledge. In Dabat district, complementary feeding practice is lower. Thus, efforts should be strengthened to boost mother’s IYCF knowledge.

## Introduction

Adequate nutrition is essential to ensure optimal health, physical and mental growth of children [1]. Complementary food, a transitional food is intended to meet the particular nutritional or physiologic needs of the young child. As a result, it is universally recommended that mothers should start nutritionally adequate and safe complementary food at the infant’s sixth month [2, 3]. Moreover, World Health Organization (WHO) supports the implementation of Infant and Young Child Feeding (IYCF) strategy for children aged 6–24 months as a critical element of efforts to address child malnutrition and mortality [2]. Hence, promotion of appropriate complementary feeding prevents 6% of child deaths in countries with high child mortality rate [4], it is of a paramount importance for developing countries, such as Africa and South-Central Asia [5]. A substantial number (2.1 million) of this mortality is contributed by undernutrition [6].

However, inappropriate complementary feeding practices have been widely documented, and stay as major public health problem in many developing countries [7]. Studies reports from Uganda [8], Ghana [9] and South Africa [10] revealed that more than half of mothers initiated complementary feeding away from sixth month. Various reports identified the determinants of timely initiation of complementary feeding practices, and it mainly related to the maternal health care utilization and socio-economic characteristics. Higher maternal educational status, unemployment [11], being married [12], having antenatal care (ANC) and home birthing [11, 12] are significantly associated with initiation of complementary feeding at the right time, sixth month. Furthermore, male sex of the child and smaller family size are related with increased odds of a timely initiation of complementary feeding [12–14]. However, lower maternal socio-economic status is inversely associated with timely initiation of complementary feeding [15].

In Ethiopia child undernutrition continues as a critical public health problem [16] and it is an underlying cause for 53% of under five child mortality [17]. Complementary feeding practice is also by far suboptimal in the country, according to which 51% of children are given complementary food at 6–9 months of age, and only 4% of young children aged 6–23 months are found with minimum dietary diversity [16]. Conducting a study in an evidence dearth setting is critical to explore information on complementary feeding practices and its determinants especially; no study is conducted in the current study area. Even the previous studies are institution based [11, 13] and done in a smaller scale, districts level [12, 18], which ultimately affects the generalizability of the finding. Therefore to fill the knowledge gap, this study aimed to determine a timely initiation of complementary feeding and associated factors among children aged 6–24 months in HDSS site. The HDSS site is established to represent the rural northwest Ethiopia.

A community-based cross-sectional study was conducted in HDSS site from 01 May to 29 June 2015. The site is located in the Dabat District, northwest Ethiopia, and hosted by the University of Gondar. The district has an estimated population size of 145,458 living in 26 rural and four urban kebeles *(the smallest administration unit Ethiopia)*. Currently, six health centers and 29 health posts are providing health services to the residents. The HDSS covers 13 randomly selected kebeles (three urban and ten rural kebeles) in different ecological zones (high land, middle land, and low land). A total of 67,385 inhabitants are living in these kebeles. The detailed data collection system, data quality control, the database, and the study setting of Dabat HDSS are described elsewhere [19].

The study is a part of the original survey which aimed to assess the nutritional status and feeding practice of children aged 6–59 months in Dabat HDSS. Of the total kebeles in the HDSS, eight kebeles were selected by using lottery method. All mothers with children aged 6–24 months and lived in the selected kebeles of the HDSS were included in the original survey. For households with multiple children, a child was selected randomly using lottery method. To estimate complementary feeding practice among mothers with children aged 6–24 months, sample size was calculated using Epi-info version 3.7 by considering the following assumptions; the prevalence of a timely initiation of complementary feeding in North Ethiopia was 62.8% [11], 95% level of confidence, 5% margin of error, 5% non-response rate, and a design effect of 1.5. Thus, a minimum sample size of 566 was obtained. However, 591 children fulfilling the inclusion criteria were found in the original survey, as a result to improve the power of the study, all the eligibles were included in the analysis.

A structured interviewer-administered questionnaire was used to collect data. The questionnaire composed of socio-demographic and economic characteristics, health care, and complementary feeding practice related variables. The questionnaire was first translated from English to Amharic, the native language of the study area, and was retranslated back to English by professional translators. Fourteen data collectors and three field supervisors were recruited for the study. Data collectors and supervisors were trained for 2 days. The tool was piloted out of the study area, thus the acceptability and applicability of the procedures and tools were evaluated. All filled questionnaires were daily checked for completeness by the supervisors and the investigators.

Complementary feeding practices were assessed according to the key indicators recommended by WHO and IYCF strategy of Ethiopia. The outcome variable, timely initiation to complementary feeding was defined as a proportion of children (6–24 months) who initiated complementary food at sixth month of age [20]. The standardized dietary diversity score tool was used to estimate feeding practice of children. To capture the usual dietary habit of children, both a single 24-h and 7 day recall methods were employed. Minimum dietary diversity was defined as proportion of children (6–24 months) who received complementary food made from four or more food groups in the previous 24-h [20]. Moreover, minimum meal frequency was determined as proportion of breastfed and non-breastfed children (6–24 months) who received solid, semi-solid or soft food for the minimum number of times or more in the 24-h preceding the survey [20].

Furthermore, the 7-day quasi food frequency, modified food group frequency was measured as the number of days the child consumed any of the seven food groups in the last 7 days preceding the date of survey. Since it shows usual dietary pattern of children, the 7-day quasi food frequency was used to overcome the limitation of 24 h recall method. A mother was asked to report for how many days her child ate any of the above seven food groups in the last 1 week. A score ranging from 1 to 7 was given depending on the number of days the child ate the listed food groups. Accordingly, if the child ate for 1–3 days it was coded as ‘1’, and 4 or more days coded as ‘2’, otherwise, the score ‘0’ was given if the child didn’t eat.

A Principal Component Analysis (PCA) was employed to estimate the mothers IYCF knowledge. Nine questions most of which are tailored from the core IYCF indicators were used to determine the mother’s knowledge. In the PCA, the knowledge item questions were summed and ranked into terciles; lowest, medium, and highest. Similarly, household wealth index was computed using a composite indicator for urban and rural residents by considering properties, like selected household assets and size of agricultural land, and PCA was performed to categorize the household wealth status into lowest, middle, and highest.

Data were entered into Epi-info version 3.5.3 and exported to Statistical Package for Social Sciences (SPSS) version 20 for analysis. Descriptive statistics, including frequencies and proportions were used to summarize variables. In order to indentify the factors associated with a timely initiation to complementary feeding, a binary logistic regression model was used. Co-linearity was checked for Household income and other independent variables like IYCF knowledge, antenatal care, and health care access and the result showed that the independent variables have no significant co-linearity. Variables with a p-values of < 0.2 in the bivariable analysis were subjected to multivariable analysis. We followed the method of Hailu et al. [21].

## Main text

**Fig. 1:**
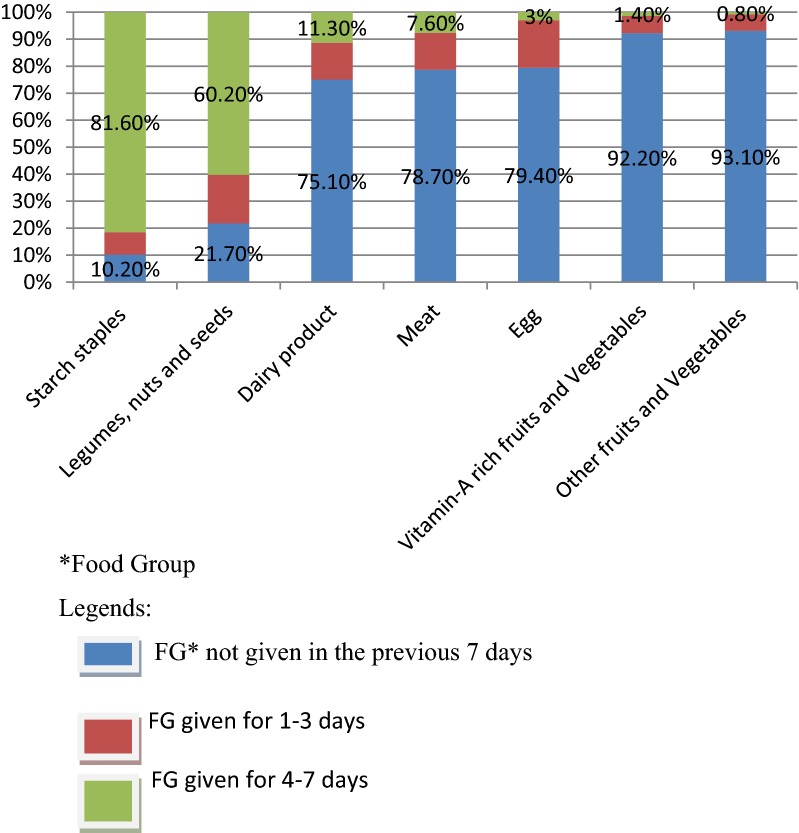
Number of days the children (6–24 months) consumed food groups in the last 7 days preceding the date of survey in the rural population of northwest Ethiopia, 2015

A total of 591 mother–child pairs were included for analysis. About two-thirds (66%) of children were in the age group of 12–24 months. More than half of mothers were housewives (59.7%) and had no formal education (70.2%). About 53.8% [95% CI 45.9, 61.7] of children were introduced to complementary feeding at their sixth month of age. Only 4.6% [95% CI 1.3, 7.9] of children consumed food composed of a minimum dietary diversity, while about 13.5% of children received the minimum meal frequency in the previous 24-h preceding the date of survey. Almost all of children didn’t eat other fruits and vegetables (98.8%), vitamin-A rich fruits and vegetables (95.9%) (Table 1). On the other hand, more than three-fourths (81.6%) and nearly two-thirds (60.2%) of children consumed starchy staples and legumes and nuts for 4–7 days per week, respectively (Fig. 1).

**Table 1:** Socio-demographic, health and dietary practice of mothers with children aged 6–24 months in the rural population of northwest Ethiopia, 2015

Characteristics	Frequency	Percent (%)
Age of child (in months)		
6–11	201	34.0
12–24	390	66.0
Age of mother (years)		
≤ 35	372	62.9
> 35	219	37.1
Marital status		
Married	532	90.0
Others^a^	59	10.0
Religion		
Orthodox Christians	552	93.4
Others^b^	39	6.6
Mothers education		
No formal education	415	70.2
Primary education	79	13.4
Secondary and above	97	16.4
Fathers education		
No formal education	396	67.0
Primary education	103	17.4
Secondary and above	92	15.6
Family size		
≤ 4	227	38.4
> 4	364	61.6
Mothers occupational status		
Housewife	353	59.7
Farmer	146	24.7
Others^c^	92	15.6
Fathers occupational status		
Unemployed	28	4.7
Farmer	172	29.1
Others^d^	391	66.2
Possession of radio or television		
Yes	98	16.6
No	493	83.4
Wealth status		
Poor	205	34.7
Medium	187	31.6
High	199	33.7
Antenatal care visits		
No antenatal care visit	208	35.2
1–3 visit	251	42.2
≥ 4 visit	132	22.3
Place of delivery		
Home	426	72.1
Health institution	165	27.9
Delivery attendant		
Health professional	168	28.6
TBA^e^	81	13.7
Relative and volunteer	341	57.7
Postnatal visit		
Yes	159	26.9
No	432	73.1
Health care access		
Good access (< 2 h)	433	73.3
Poor access (> 2 h)	158	26.7
Mothers IYCF knowledge		
Poor	187	31.6
Medium	209	35.4
High	195	33.0
Food groups		
Starchy staples	502	84.9
Legumes and nuts	361	61.1
Dairy products	106	17.9
Flesh food (meat)	59	10.0
Eggs	39	6.6
Vitamin-A rich fruits and vegetables	24	4.1
Other fruits and vegetables	7	1.2

^a^Single, divorced and widowed

^b^Muslim, protestant and catholic

^c^Students, unemployed, servant, own business

^d^Contract and permanent work

^e^Traditional birth attendant

The result of the multivariable logistic regression analysis showed that mothers IYCF knowledge was significantly and independently associated with timely initiation to complementary feeding. Accordingly, the odds of timely initiation of complementary feeding were higher among mothers with medium [AOR = 2.34, 95% CI 1.54, 3.81] and high [AOR = 2.10, 95% CI 1.41, 3.87] IYCF knowledge compared to mothers with poor IYCF knowledge (Table 2).

**Table 2:** Factors associated with timely initiation of complementary feeding in the rural population of northwest Ethiopia, 2015

Characteristics	Timely initiation of complementary feeding		Crude odds ratio (95% CI)	Adjusted odds ratio (95% CI)
	Yes (#)	No (#)		
Initiation of breastfeeding				
Early initiation	36	29	1.08 (0.64, 1.8)	
Late initiation	282	244	1	
Mothers IYCF knowledge				
Poor	113	74	1	1
Medium	77	132	2.62 (1.74, 3.93)	2.34 (1.54, 3.81)*
High	83	112	2.06 (1.37, 3.10)	2.10 (1.41, 3.87)*
ANC visits				
No	121	87	1	
1–3	130	121	0.65 (0.41, 1.02)	
≥ 4	67	65	0.82 (0.57, 1.18)	
Health care access				
Good access	216	217	0.55 (0.38, 0.8)	
Poor access	102	56	1	
Wealth status				
Poor	101	104	1	
Medium	112	75	1.54 (1.03, 2.30)	
High	105	94	1.15 (0.78, 1.70)	
Place of delivery				
Home	234	192	1	
Health institution	84	81	0.85 (0.59, 1.22)	
Postnatal visit				
Yes	86	73	1.02 (0.71, 1.43)	
No	232	200	1	
Mothers educational status				
No formal education	217	198	1	
Primary education	44	35	1.15 (0.71, 1.86)	
Secondary and above	57	40	1.30 (0.83, 2.04)	

* Significant at p < 0.05

The prevalence of timely initiation of complementary feeding was 53.8%, and it was consistent with other study findings in Ethiopia (51–62.8%) [11–13, 16] and India (55.1%) [22]. However, it was lower than reports of other developing countries, such as Nepal (70%) [23], Bangladesh (71%) [24], and Sir Lanka (84%) [25]. The observed discrepancy probably relates to difference in place of residence among the study participants and almost all of the mothers were rural inhabitants in this study. There has been lesser access to education, health care services, and other resources (printed and electronic medias) in the rural settlements [16]. These social inequalities lower the maternal literacy status and access to nutrition education and counseling. In fact improved literacy status and nutrition education are found to positively affect the complementary feeding practices [26, 27]. Furthermore, the discrepancy could be attributed to variation in the measurement of initiation of complementary feeding, in which the latter studies assessed initiation of complementary food at 6–8 months of age of the child while it was at sixth month for the former study. Thus, use of the reference period 6–8 months might over estimated the prevalence.

The odds of timely initiation of complementary feeding was higher among mothers with medium and higher IYCF knowledge. This finding was supported with another report in East Ethiopia [17]. The plausible explanation could be related to the role of knowledge to empower mother’s to resist external interferences and pressures from traditional faiths and misunderstandings favoring untimely initiation of complementary feeding. Improved health care access gives an added opportunity for mothers to get nutritional counseling thereby to practice the optimal IYCF [28]. The study finding could support to strengthen nutrition education through tailoring with behavioral change and communication components to address the community misconceptions towards IYCF practice.

The prevalence of minimum acceptable dietary diversity was very low (4.6%), and it was similar with what was reported from Amhara region, northwest Ethiopia (2%) [18]. However, the finding was lower than the reports from other developing countries, like India (15.2%) [29], Nepal (34%) [23], Bangladesh (41.9%) [24], and Sri Lanka (71%) [25]. This is probably related to lower economic status of the households in the study area, which impairs the households to purchase and offer diversified food to their child. Other studies also noted that, the households socio-economic status and per capita food expenditure was positively associated with dietary diversity [30–32]. In addition, the lower dietary diversity could be due to the monotonous dietary habit of the community in this study area. In Ethiopia including the current study area, complementary food are made from cereals which is usually served with legumes or pulses [16]. In this study, the prevalence of timely initiation of complementary feeding and dietary diversity was low. Furthermore, mothers’ IYCF knowledge and health care access were significantly associated with timely initiation of complementary feeding. Thus, efforts should be strengthened to boost mothers’ IYCF knowledge.

## Limitations

As an illustration the cross sectional nature of the study limits measuring the cause and effect relationship between the potential factors and the outcome. Also there might be a recall and social desirability bias while subjects were requested to give dietary information.

## Abbreviations

PCA: Principal Component Analysis
WHO: World Health Organization
ANC: antenatal care
AOR: adjusted odds ratio
COR: crude odds ratio
CI: confidence interval
IYCF: Infant and Young Child Feeding
DDS: dietary diversity score
SD: standard deviation
HDSS: Health and Demographic Surveillance System
